# A novel prognostic signature based on mitochondrial permeability transition-driven necrosis genes for biochemical recurrence prediction in prostate cancer

**DOI:** 10.3389/fonc.2026.1775602

**Published:** 2026-04-02

**Authors:** Xing Luo, Zeyu Huang, Ming Deng, Jinggui Liu, Bishao Sun, Jingzheng Zhu, Yu Chen, Shuai Su, Jiang Zhao, Ji Zheng

**Affiliations:** 1 Department of Urology, Urologic Surgery Center, Xinqiao Hospital, Third Military Medical University (Army Medical University), Chongqing, China; 2 School of Medicine, Chongqing University, Chongqing, China; 3 State Key Laboratory of Trauma and Chemical Poisoning, Third Military Medical University (Army Medical University), Chongqing, China

**Keywords:** biochemical recurrence-free survival, mitochondrial permeability transition-driven necrosis, prognostic genes, prostate adenocarcinoma, risk score

## Abstract

**Background:**

Mitochondrial permeability transition-driven necrosis (MPT-DN) is a therapeutic target and critical driver of prostate adenocarcinoma (PRAD) progression. We investigated MPT-DN-related prognostic features in PRAD.

**Methods:**

PRAD transcriptomics and MPT-DN-RGs were sourced from public databases. WGCNA, differential expression, Cox regression, and machine learning identified BCR-FS prognostic genes. These genes built a risk model, revealing independent prognostic factors. Patients were stratified into high/low-risk groups. Pathways, immune microenvironment, and drug sensitivities were analyzed between groups. Finally, protein expression was validated in PCa versus normal tissues.

**Results:**

TREM2, FNDC1, and S100A8 were identified as prognostic genes. The developed risk model demonstrated strong predictive capabilities in BCR-FS, and subsequent analysis confirmed risk score, Gleason, T stage, and prostate specific antigen (PSA) as independent prognostic factors. The majority of the enrichment pathways in the high-risk group (HRG) and low-risk group (LRG) were related to the metabolism. Moreover, it was found that HRG and LRG displayed distinct immune landscapes, with HRG exhibiting immune exclusion and stronger immune evasion capabilities. Lastly, analysis of drug sensitivity showed significant differences for 6 drugs, with all values being lower in the HRG.

**Conclusion:**

This study identified TREM2, FNDC1, and S100A8 as key MPT-driven necrosis-related genes predicting biochemical recurrence in PRAD. The risk model effectively stratified patients, revealing immune exclusion and drug resistance in high-risk cases, offering prognostic and therapeutic insights.

## 1 Introduction

Prostate adenocarcinoma (PRAD) is one of the most common malignancies in men worldwide, currently ranking as the second most prevalent cancer globally among males. In 2022, there were approximately 1.41 million new cases, accounting for 14% of all male cancer cases (1). Its incidence and mortality rates remain alarmingly high, posing a significant threat to men’s health. Advances in early screening and diagnostic technologies have led to increased detection of prostate cancer at early stages. In Europe and North America, prostate cancer dominates male cancer statistics, representing 30% of all male cancer cases and ranking first in incidence (2). For clinically localized or intermediate-risk prostate cancer, the primary treatments include radical prostatectomy and radical radiotherapy (3, 4). However, despite curative-intent therapies, a substantial proportion of patients experience biochemical recurrence (BCR), characterized by a rise in prostate-specific antigen (PSA) levels, which often heralds disease progression and poor prognosis (3). Therefore, accurate prediction of BCR risk is critical for tailoring individualized treatment strategies and improving patient outcomes.

Mitochondrial permeability transition-driven necrosis (MPT-DN) is a caspase-independent cell death modality triggered by aberrant opening of the mitochondrial permeability transition pore (mPTP) (5, 6). Emerging evidence highlights its pivotal role in diverse pathological contexts, including cancer. During oncogenesis and tumor progression, MPT-DN dynamically regulates tumor cell survival and death, thereby influencing disease progression and clinical outcomes (6–8). Notably, activation of the MPT-DN pathway correlates with cytotoxicity induced by certain chemotherapeutic agents, directly linking this process to tumor cell demise (9). Furthermore, MPT-DN may critically impact tumor prognosis through modulation of metabolic reprogramming, immune microenvironment alterations, and therapeutic sensitivity (6, 8). Although direct associations between MPT-DN-related genes (MPT-DN-RGs) and prostate cancer remain underexplored, accumulating data underscore the functional relevance of mitochondrial dysfunction in prostate carcinogenesis. For instance, mitochondrial biomarker expression in prostate cancer tissues correlates with tumor grade and staging, suggesting mitochondria-related genes as potential prognostic indicators (10, 11). Consequently, delineating the mechanistic contributions of MPT-DN-RGs in prostate cancer is imperative for elucidating disease pathophysiology and advancing prognostic precision.

This study aims to investigate the prognostic significance of mitochondrial permeability transition-driven necrosis (MPT-DN) related genes in prostate adenocarcinoma (PRAD) by identifying key genes associated with biochemical recurrence (BCR) and developing a risk assessment model. Through systematic bioinformatics analysis, we identified three prognostic genes and constructed a nomogram to predict BCR risk in PRAD patients. Additionally, we explored biological differences between high-risk and low-risk groups and validated protein expression levels of these prognostic genes.

## 2 Materials and methods

### 2.1 Data source

The datasets related to prostate adenocarcinoma (PRAD) included in this study were all sourced from public databases. Specifically, RNA sequencing (RNA-seq) data, survival information (overall survival (OS), disease-free survival (DFS), relapse-free survival (RFS), progression-free survival (PFS), and biochemical recurrence-free survival (BCR-FS)), and clinical characteristics for PRAD were downloaded from TCGA database (https://portal.gdc.cancer.gov/) (TCGA-PRAD), which served as the training cohort (12). This dataset included samples of 481 PRAD tumor tissues and 51 control tissues, with biochemical recurrence (BCR) information recorded in 414 of the PRAD samples. Among these, 396 samples contained follow-up time, allowing for classification based on BCR status: 50 samples were identified as BCR (indicating recurrence), while 346 samples were no-BCR (indicating no recurrence). An additional PRAD dataset was downloaded from the GEO database (https://www.ncbi.nlm.nih.gov/geo/) as a validation cohort, identified as GSE70768, which was based on the GPL10558 platform. This dataset, originally comprising RNA-seq data from 125 PRAD tumor tissue samples, was reduced to 111 samples after excluding those lacking BCR-FS information for analysis (13). Clinical metadata, including BCR status and time-to-event, were harmonized across the TCGA-PRAD and GSE70768 cohorts. Following the AUA 2019 guidelines, BCR was defined as two consecutive PSA levels ≥ 0.2 ng/mL recorded at least three months apart post-radical prostatectomy. The TCGA training cohort (n=396) recorded a 12.6% BCR rate over a median 24-month follow-up, while the GSE70768 validation cohort (n=111) exhibited a ~30% BCR rate over a median 72-month period. Both cohorts demonstrated comparable Gleason score distributions (6–10) and treatment homogeneity, with radical surgery serving as the primary intervention (>95% in TCGA). This consistency in clinical management and diagnostic criteria minimizes potential confounding effects on prognostic outcomes. Furthermore, mitochondrial permeability transition (MPT)-driven necrosis-related genes (MPT-DN-RGs) were sourced from the gene set enrichment analysis (GSEA) database (https://www.gsea-msigdb.org/gsea), specifically pathways numbers Go Mitochondrial_Permeability_Transition (m17902), Reactome_Mitochondrial_Membrane_Permeabilization (m3873), and Wp_Mitochondrial_Dysfunction (m16257). These gene sets encompass genes functionally involved in mPTP opening, cyclophilin D–dependent signaling, and mitochondrial stress responses, which represent the core molecular mechanisms underlying MPT-driven necrosis (14). Subsequently, the genes obtained from each gene set were combined, and duplicates were eliminated, resulting in a total of 39 MPT-DN-RGs for this study (**Supplementary Table 1**).

### 2.2 Acquisition of genes associated with MPT-DN-RGs

To explore the genes associated with MPT-DN-RGs in the 414 PRAD samples with BCR information, single-sample GSEA (ssGSEA) algorithm from GSVA (v 1.48.3) package (15) was employed to evaluate scores for each sample of the 414 PRAD samples with respect to MPT-DN-RGs. Following this, MPT-DN-RGs scores were integrated as a trait with gene expression data in the 414 PRAD samples to construct a co-expression network using weighted gene co-expression network analysis (WGCNA). The WGCNA (v 1.71) package (16) was employed for identifying modules most correlated with MPT-DN-RGs scores. All the 414 PRAD samples with BCR information were clustered, and outliers were removed. To ensure that the interactions between genes closely followed a scale-free distribution, a scale-free fit index R^2^ approaching 0.8 was set, while maintaining mean connectivity approached zero, thus determining optimal soft-threshold (power). Genes were then merged at the minimum number of genes in per gene module of 150 to yield modules relevant to MPT-DN-RGs scores. After module detection, a dendrogram of gene similarity was plotted, and the correlation analysis between these modules and MPT-DN-RGs scores was calculated with Pearson, followed by the creation of a corresponding heatmap to visualize the correlations. The module exhibiting the highest correlation with MPT-DN-RGs scores was selected as the key module (|correlation coefficient (cor)| > 0.3, p*<* 0.05). Genes within the key module were further considered as genes associated with MPT-DN-RGs.

### 2.3 Identification and enrichment analysis of candidate genes

In the training cohort, differential expression analysis was performed using DESeq2 (v 1.40.2) package (17) between PRAD and control samples (|log_2_Fold Change (FC)| > 0.5, p< 0.05), referred to as DEGs1. Subsequently, a similar analysis was performed to identify DEGs between BCR samples and no-BCR samples, referred to as DEGs2, applying the same thresholds. To visualize the distribution and expression of both DEGs, volcano plots and heatmaps were created employing ggplot2 (v 3.4.2) (18) and pheatmap (v 1.0.12) packages (19), respectively. The top 10 up- and down-regulated genes of both DEGs were separately labelled in the volcano plots according to their log_2_FC.

Further analysis involved taking the intersection of DEGs1, DEGs2, and genes associated with MPT-DN-RGs to identify candidate genes, which were visualized operating eulerr (v 7.0.0) package (20). To understand the potential biological functions and signaling pathways associated with candidate genes, clusterProfiler (v 4.8.2) package (21) was utilized to carry out Gene Ontology (GO) and Kyoto Encyclopedia of Genes and Genomes (KEGG) enrichment analyses for candidate genes. The raw P-values of all enriched pathways were adjusted using the Benjamini–Hochberg (BH) method to control the false discovery rate (FDR), and pathways with adjusted P-values (Padj)< 0.05 were considered statistically significant.

### 2.4 Recognition of the prognostic genes

Within the 396 samples in the training cohort that contained follow-up durations, candidate genes were subjected to univariate Cox regression analysis by survival (v 3.5.5) package (22), with selection criteria set at hazard ratio (HR) ≠ 1 and p< 0.05. Visualization was achieved through forest plots generated with the forestplot (v 2.0.1) package (23). Genes that passed the screening were further tested for the proportional hazards (PH) assumption, and those meeting the PH assumption criteria were selected as BCR-FS related genes (p > 0.05). With the help of glmnet (v 4.1.6) package (24), the least absolute selection and shrinkage operator (LASSO) regression analysis was performed with 10-fold cross-validation. Genes identified by LASSO were further tested underwent multivariate Cox regression analysis *via* survival (v 3.5.5) package (HR≠1, p< 0.05). The model was optimized using stepwise regression, ultimately identifying prognostic genes and their corresponding risk coefficients for this study.

Furthermore, the 396 PRAD samples were categorized into groups of high- and low-expression by utilizing median values derived from the expression levels of prognostic genes, respectively. The Kaplan-Meier (K-M) survival analysis for each gene was then exploited using survminer (v 0.4.9) package (25) to separately compare the differences in sample survival (OS, DFS, RFS, and PFS) between the high- and low-expression groups (p< 0.05).

### 2.5 Construction and validation of risk model

Subsequent analysis aimed to evaluate the value of prognosis genes for the prognosis of BCR-FS in PRAD samples. Among the 396 PRAD samples of the training cohort, according to the expression levels of previously identified prognostic genes and their corresponding risk coefficients from multivariate Cox regression analysis, a risk score for each PRAD patient was calculated. The formula was


$$risk\;score=\sum_{i=1}^{n}\left(coefi*Xi\right)$$

where Xi represented the expression level of prognostic genes, normalized using a $\log_{2}(FPKM+1)$ transformation, and coefi denoted the risk coefficient of the corresponding gene (the specific regression coefficients used in the final model are presented in the Results section). Under these circumstances, patients were divided into high-risk and low-risk groups (HRG and LRG) *via* median value of the risk scores. Risk curve was then plotted to display the distribution of risk scores and survival status among PRAD patients at different risk levels, and heatmap was generated to show the expression trends of the prognostic genes across the 2 risk stratifications. Moreover, K-M survival curve was drawn to assess the BCR-FS of patients in different risk strata by survminer (v 0.4.9) package (p< 0.05), and receiver operating characteristic (ROC) curves were constructed through timeROC (v 0.4) package (26) to quantify the area under curves (AUCs) of the risk model at 1, 3, and 5 year. In a similar manner, the risk model was validated in the validation cohort GSE70768.

### 2.6 Assessment of risk score association with clinical characteristics

Utilizing a background of 396 samples with BCR-FS information in the training cohort, risk scores were analyzed in conjunction with the clinical characteristics of PRAD patients, including age, Gleason, prostate specific antigen (PSA), and T stages, to elucidate the predictive capability of risk scores for PRAD patient characteristics. Specifically, the differences in risk scores across different clinical subtypes were examined (p< 0.05). Furthermore, the distribution proportions of clinical characteristics among HRG and LRG were compared utilizing Chi-square test (p< 0.05).

### 2.7 Independent prognostic analysis and construction of nomogram

Risk scores and clinical characteristics of PRAD patients were sequentially integrated into univariate Cox regression (HR≠1, p< 0.05), and then included in PH assumption test (p > 0.05) and multivariate Cox regression (HR≠1, p< 0.05). This approach enabled the identification of independent prognostic factors for PRAD. These independent prognostic factors were then incorporated into a nomogram within the training cohort by rms (v 6.7.0) package (27) to predict the 1-, 3-, and 5-year BCR-FS probabilities of PRAD patients. In that case, to evaluate the predictive accuracy of the nomogram, calibration curves were drawn employing calibrate (v 1.7.7) package (28), as well as decision curve analyses (DCA) were conducted using the rmda (v 1.6) package (29).

### 2.8 Functional and annotation analysis

In order to elucidate the pathways associated with both risk groups, in the 396 PRAD samples of the training cohort, an initial analysis of differential gene expression was performed utilizing DESeq2 (v 1.40.2) package, with genes ranked by their log_2_FC. Then, GSEA was conducted by clusterProfiler package (v 4.8.2) (adj.p< 0.05) on rank results. The reference gene sets were “c2.cp.kegg.v2023.1.Hs.symbols” downloaded from Molecular Signatures Database (MSigDB) (https://www.gsea-msigdb.org/gsea/msigdb), and the top 5 enriched pathways were visualized. Subsequently, to further explore the differences in pathways involved between HRG and LRG, the GSVA (v 1.48.3) package was used to perform gene set variation analysis (GSVA) applying “c2.cp.kegg.v2023.1.Hs.symbols” background gene set from MSigDB. Enrichment scores for various pathways were obtained, and functional enrichment pathways between HRG and LRG (adj.p< 0.05) were compared *via* limma (v 3.56.2) package (30). The top 10 up- and down-regulated pathways were visualized, ranked by their log_2_FC, using a heatmap generated with the ggplot2 (v 3.4.2) package.

### 2.9 Immune landscape analysis

Further examination focused on the comparative evaluation of immune cell infiltration levels within immune microenvironments of HRG and LRG. Leveraging the computational methodology of ssGSEA algorithm from GSVA (v 1.48.3) package, an in-depth analysis was conducted to assess the infiltration of 28 distinct immune cells across HRG and LRG (31). The differences in immune cells infiltration between HRG and LRG were examined to identify differential immune infiltrating cells (p< 0.05), with the results visualised in a box plot by ggplot2 (v 3.4.2) package. Moreover, pearson correlation analyses were constructed *via* psych (v 2.3.6) package to investigate potential correlations between differential immune infiltrating cells with noteworthy differences as well as between such immune cells and prognostic genes(|cor| > 0.3, p< 0.05) (32).

In an effort to gauge potential clinical efficacy of immunotherapy for patients stratified by risk, the Tumor Immune Dysfunction and Exclusion (TIDE), dysfunction, microsatellite instability (MSI), and exclusion scores were calculated in HRG and LRG through TIDE database (http://tide.dfci.harvard.edu/). This analysis allowed us to evaluate the variance in these scores betwixt HRG and LRG (p< 0.05), offering insights into the immunotherapy response potential across different risk groups. In addition, a comparative analysis of 11 prototypical immune checkpoint genes was conducted: CD274, PDCD1, CTLA4, TIGIT, LAG3, LGALS9, HAVCR2, PDCD1LG2, SIRPA, BTLA, and SIGLEC7. Within the HRG and LRG, seeking to identify differential expression patterns of these immune checkpoint genes between the risk groups (p< 0.05).

### 2.10 Analysis of mutated landscapes and chemotherapeutic drug sensitivity

To investigate the genetic differences between HRG and LRG in the 396 PRAD samples from the training cohort, the maftools (v 2.16.0) package (33) was employed to analyze 2 risk groups of patients with mutation data. After that, the top 20 frequency mutated genes were visualized in a waterfall plot, while the association between risk score and tumor mutational burden (TMB) was also investigated *via* psych (v 2.3.6) package (|r| > 0.3, p< 0.05). Additionally, the 396 PRAD samples were categorized into high- and low-TMB groups based on the median TMB score. The HRG and LRG were then combined with the high- and low-TMB groups, and the K-M survival analyses for each combination were then exploited using survminer (v 0.4.9) package to compare the differences in BCR-FS among these groups (p< 0.05).

With a view to explore the potential application of common chemotherapy drugs in patients with different risk stratifications, 198 common chemotherapy drugs (34) were obtained from Genomics of Drug Sensitivity in Cancer (GDSC) database (http://cancerrxgene.org). The oncoPredict (v 0.2) package (35) was utilized to obtain half maximal inhibitory concentration (IC_50_) values for 396 PRAD samples in the training set, and the IC_50_ between HRG and LRG for each chemotherapeutic drug was compared (p< 0.05). To elucidate these findings, the ggplot2 (v 3.4.2) package was utilized to graphically represent these drugs with the pronounced differences in IC_50_ values through box plots.

### 2.11 Expression analysis of prognostic genes

To observe the expression differences of prognostic genes between PRAD and control samples, the differential expressions of prognostic genes were visualized in the training set of PRAD and control samples (p< 0.05), followed by the generation of violin plot for visualization. Subsequently, immunohistochemical images and corresponding data were obtained from the Human Protein Atlas (HPA) database (https://www.proteinatlas.org) to examine the protein expression levels of prognostic genes in both PRAD and control tissue samples.

### 2.12 Clinical sample collection

Between January 2024 and December 2024, ten paired PRAD tumor tissues and adjacent non-cancerous tissues were prospectively collected from the Second Affiliated Hospital of Army Medical University (AMU). Clinical sample collection: These patients were representative of typical localized prostate adenocarcinoma (PRAD) cases that underwent radical prostatectomy. They were characterized by organ-confined or locally advanced disease (pathological stage T2–T3), intermediate- to high-grade tumors (Grade Group 2–4), and a median preoperative PSA of 17.2 ng/mL (range: 7.9–40.3). These specimens were utilized for Western blot and immunohistochemical (IHC) analyses. Prostate cancer tissues were surgically obtained from patients undergoing radical prostatectomy. All tissues underwent histopathological confirmation by at least two board-certified pathologists following standard hematoxylin and eosin (H&E) staining protocols. This study was approved by the Institutional Review Board (IRB) of the Second Affiliated Hospital of AMU (approval no. 2024-193-02) and adhered to the ethical principles outlined in the Declaration of Helsinki. Written informed consent was obtained from all study participants or their legally authorized representatives prior to tissue procurement.

### 2.13 Western blot

Western blotting experimental methods were consistent with those previously reported (36). For electrophoresis, 40-μg protein was used. The following were the antibody concentrations for western blotting: rabbit polyclonal anti-FNDC1 antibody (1:1000, catalog no. bs-8460R; Bioss, Beijing, China), rabbit polyclonal anti-TREM2 antibody (1:1000, catalog no. HY-P80920; MedChemExpress, Monmouth Junction, NJ, USA), and rabbit polyclonal anti-S100A8 antibody (1:1000, catalog no. AF7929; Beyotime, Shanghai, China), and mouse monoclonal anti-GAPDH (1:2000, 60004-1-Ig, Proteintech, China).

### 2.14 Quantitative real-time polymerase chain reaction

10 paired PRAD tumor tissues and adjacent non-cancerous tissues were prospectively collected. Quantitative real-time PCR was performed as previously reported (37). The primer sequences are provided as follows:

GAPDH-Forward:5′-TCAAGAAGGTGGTGAAGCAGG-3′,

GAPDH-Reverse:5′- TCAAAGGTGGAGGAGTGGGT -3′;

FNDC1-Forward: 5′-CAGTAAGGCGGATGTTGAGC-3′,

FNDC1-Reverse:5′-TTGGGGAGAAGCAGGCAC-3′; S100A8-Forward:5’-TGGCTCTCCTTCTCCTCTTC-3’,

S100A8-Reverse: 5’-GCAGGATCTTGGTGTTGGTT-3’

,TREM2-Forward:5’-CTGCTGGTGCTGATGGTATT-3’,

TREM2-Reverse: 5’-GGAGCTGGTGGTAGTGATGG-3’.

### 2.15 Immunohistochemical staining

Formalin-fixed paraffin-embedded (FFPE) prostate tissue specimens were processed for IHC analysis. IHC staining protocols strictly adhered to our previously validated methodologies (38), which included antigen retrieval using citrate buffer (pH 6.0), peroxidase blocking, and 3,3’-diaminobenzidine (DAB) chromogen development. All slides were independently evaluated by two board-certified pathologists blinded to clinical data, with discrepancies resolved by consensus review. The following primary antibodies were utilized at optimized dilutions: rabbit polyclonal anti-FNDC1 antibody (1:1000, catalog no. bs-8460R; Bioss, Beijing, China), rabbit polyclonal anti-TREM2 antibody (1:1000, catalog no. HY-P80920; MedChemExpress, Monmouth Junction, NJ, USA), and rabbit polyclonal anti-S100A8 antibody (1:1000, catalog no. AF7929; Beyotime, Shanghai, China). All antibodies were validated for specificity in prior studies through western blot confirmation and isotype-matched controls.

### 2.15 Statistical analysis

All statistical analyses were conducted utilizing the R (v 4.2.2) language. To determine whether there were statistical differences between the 2 groups, the Wilcoxon test and log-rank test were employed, while the Kruskal-Wallis test was used for comparisons among multiple groups. A p value of less than 0.05 was considered to be statistically significant.

## 3 Results

### 3.1 Identification of 1,199 genes associated with MPT-DN-RGs

**Figure 1:**
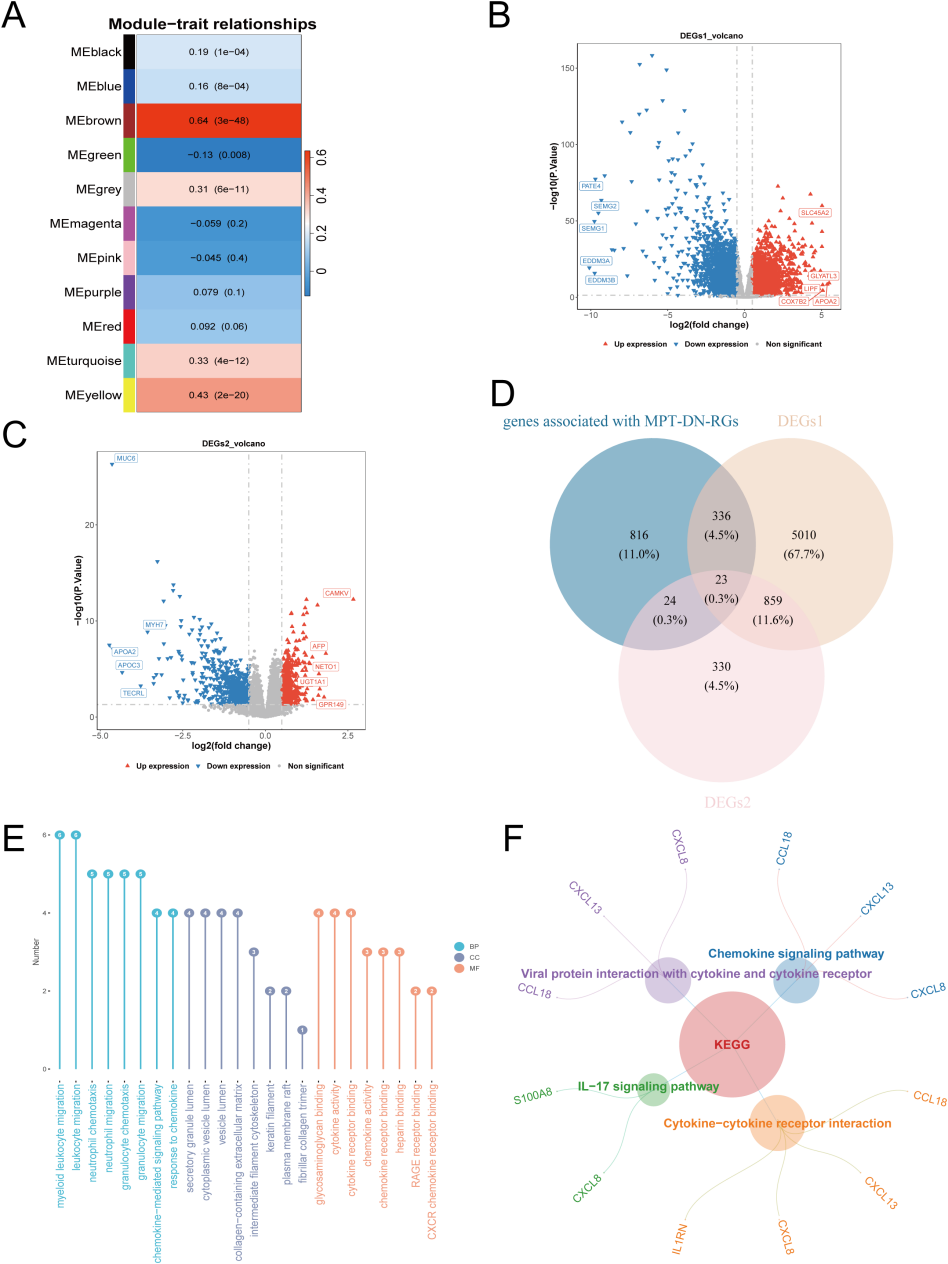
Identification of MPT-DN-RGs-Related candidate genes and enrichment analysis. **(A)** Correlations of gene modules with MPT-DN-RGs scores. **(B)** Volcano plot of differentially expressed genes between PRAD tumor and control tissues. **(C)** Volcano plot of differentially expressed genes between BCR samples and no-BCR samples. **(D)** Venn diagram of overlapping genes in PRAD, BCR and MPT-DN-RGs-related genes. **(E)** GO enrichment analysis of the 23 candidate genes, showing the top enriched terms in biological processes (BP), cellular components (CC), and molecular functions (MF). **(F)** KKEGG pathway enrichment analysis of the 23 candidate genes, presenting the significantly enriched pathways (p < 0.05).

To the beginning, MPT-DN-RGs scores were utilized as a trait for WGCNA which aimed at identifying key module with MPT-DN-RGs, and genes associated with MPT-DN-RGs were later identified based on key module. Specifically, clustering of the samples indicated no outliers in the 414 PRAD samples with BCR information (**Supplementary Figure 1A**). When scale-free fit index R^2^ reached 0.8 and mean connectivity value was close to zero, an optimal soft-threshold (power) of 6 was determined (**Supplementary Figure 1B**). By setting the minModuleSize to 150, 11 modules were found in total, including a grey module that could not be classified (**Supplementary Figure 1C**). The MEbrown module exhibited the strongest correlation with MPT-DN-RGs scores (cor = 0.64, p< 0.0001), which was designated as the key module, comprising 1,199 genes associated with MPT-DN-RGs that were carried forward for subsequent analysis (**Figure 1A**).

### 3.2 Recognition and enrichment analysis of 23 candidate genes

After differential expression analysis between 481 PRAD tumor tissues and 51 control tissues in training cohort, 6,228 DEGs1 were identified between PRAD and control samples, comprising 2,886 up-regulated and 3,342 down-regulated genes (**Figure 1B**; **Supplementary Figure 1D**). Similarly, the differential expression analysis among 50 BCR samples and no-BCR 346 samples uncovered 1,236 DEGs2, including 480 up-regulated genes and 756 down-regulated genes (**Figure 1C**; **Supplementary Figure 1E**). Intersecting the 1,199 genes associated with MPT-DN-RGs, 6,228 DEGs1, and 1,236 DEGs2 yielded 23 candidate genes (**Figure 1D**). Subsequently, the 23 candidate genes were significantly enriched in the 430 GO terms, including 358 biological processes (BPs), 19 cellular components (CCs), and 53 molecular functions (MFs) (**Figures 1E****;** **Supplementary Table 2**). At the GO level, these candidate genes were primarily associated with functions like “neutrophil chemotaxis” and “chemokine activity”. Further analysis revealed that the 23 candidate genes were predominantly involved in 4 KEGG pathways, encompassing “chemokine signaling pathway” and “IL-17 signaling pathway” (**Figures 1F****;** **Supplementary Table 2**). These pathways were related to inflammatory responses and immunologic system, providing a significant foundation to understand the functional implications of candidate genes in the progression of PRAD.

### 3.3 Selection of TREM2, FNDC1, and S100A8 as prognostic genes

**Figure 2:**
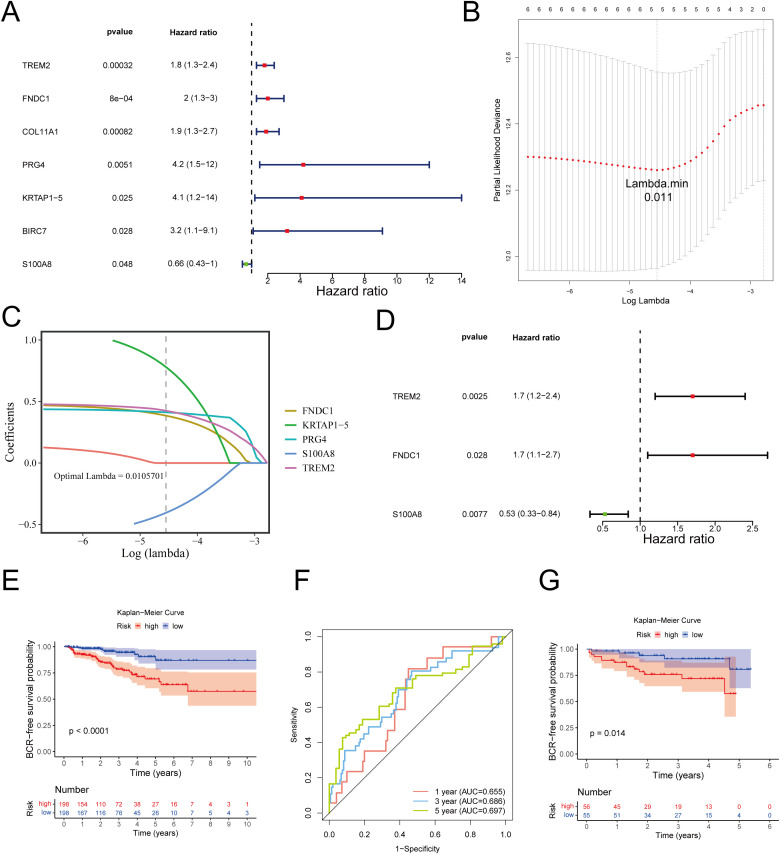
Construction of MPT-DN-RGs-related prognostic risk model. **(A)** Forest plot of univariate cox regression analysis. **(B)** Scatter plot of the minimum lambda in the lasso model using 10 folds of cross-validation. **(C)** Coefficient distribution plot of LASSO regression. **(D)** Forest plot of multivariate cox regression analysis. **(E)** Kaplan-Meier survival curves of two groups divided by median risk score in TCGA train set. **(F)** Time‐dependent ROC curve analysis of risk score at 1-, 3-, and 5-years BCR-FS time in TCGA train set. **(G)** Kaplan-Meier survival curves of two groups divided by median risk score in GSE70768 validation set.

Among the 396 samples with follow-up durations in the training cohort, the 23 candidate genes were submitted to univariate Cox regression analysis (HR ≠ 1, p< 0.05) and subsequently included in PH assumption test (p > 0.05), resulting in the identification of 7 BRC-FS related genes: TREM2, FNDC1, COL11A1, PRG4, KRTAP1-5, BIRC7, and S100A8 (**Figures 2A**; **Supplementary Table 3**). Following this, LASSO regression analysis was used to screen out 5 genes (TREM2, FNDC1, PRG4, KRTAP1-5, and S100A8) with lambda.min of 0.0105701 (**Figures 2B, C**; **Supplementary Table 3**). Furthermore, the results of multivariate Cox regression analysis were adjusted through stepwise regression approach to optimize the model, confirming 3 prognostic genes: TREM2, FNDC1, and S100A8 (**Figure 2D**), and the risk coefficients of these 3 genes were determined through multivariate Cox regression analysis (**Supplementary Table 3**). Further KM survival analyses revealed that a significant difference in RFS was observed between high- and low-expression groups of FNDC1, while significant differences in both DFS and RFS were noted for TREM2 (**Supplementary Figure 2**).

### 3.4 Robust predictive ability of the risk model

Capitalizing on the above data, a risk score equation was formulated: risk score = 0.5252 × TREM2 + 0.5338 × FNDC1 + (-0.6393) × S100A8, offering a quantitative tool for BCR-FS prognostication in PRAD. According to median value for the risk score (0.7033 for the training cohort, 2.7243 for the validation cohort), 396 PRAD patients with BCR-FS information were divided into 198 HRG and 198 LRG in the training cohort, as well as 111 PRAD patients were stratified into 56 HRG and 55 LRG in the validation cohort. The distribution of risk scores, survival status across distinct risk groups for 396 PRAD patients in training cohort were depicted in **Supplementary Figure 3A**. Heatmap displayed the expression of prognostic genes across risk categories, revealing TREM2 and FNDC1 had higher expressions of in HRG, while S100A8 was more expressed in LRG (**Supplementary Figure 3B**). K-M survival analysis confirmed that, as expected, the patients in the HRG had poorer BCR-FS (p< 0.0001) (**Figure 2E**). The risk model’s ROC curves, showing AUCs of 0.655, 0.686, and 0.697 at 1-, 3-, and 5-year, respectively, suggested that the risk model possessed a reasonable predictive accuracy (**Figure 2F**). Besides, validation of the risk model in GSE70768 dataset confirmed its robustness (**Figure 2G**), as evidenced by AUCs exceeding 0.6 for 1-, 3-, and 5-year, underscoring the model’s consistent prognostic strength (**Supplementary Figures 3C–E**). To evaluate its prognostic performance, the MPT-DN signature was compared with four established PRAD BCR models (ranging from 1 to 13 genes) (**Supplementary Table 7**). In the GSE70768 cohort, the MPT-DN model achieved the highest C-index (0.741) and superior time-dependent AUCs at 1, 3, and 5 years (**Supplementary Table 7**; **Supplementary Figure 5A**). Despite its simplicity (only three genes), it outperformed or matched models with 7–13 genes, suggesting enhanced clinical feasibility. Furthermore, Decision Curve Analysis (DCA) demonstrated that the MPT-DN signature consistently provided the highest clinical net benefit across all threshold probabilities (10%, 20%, and 30%), significantly surpassing the reference models (**Supplementary Table 7**; **Supplementary Figure 5B**). These results confirm the superior predictive accuracy and clinical utility of the MPT-DN model.

### 3.5 Evaluating the predictive capability of risk scores for clinical characteristics

**Figure 3:**
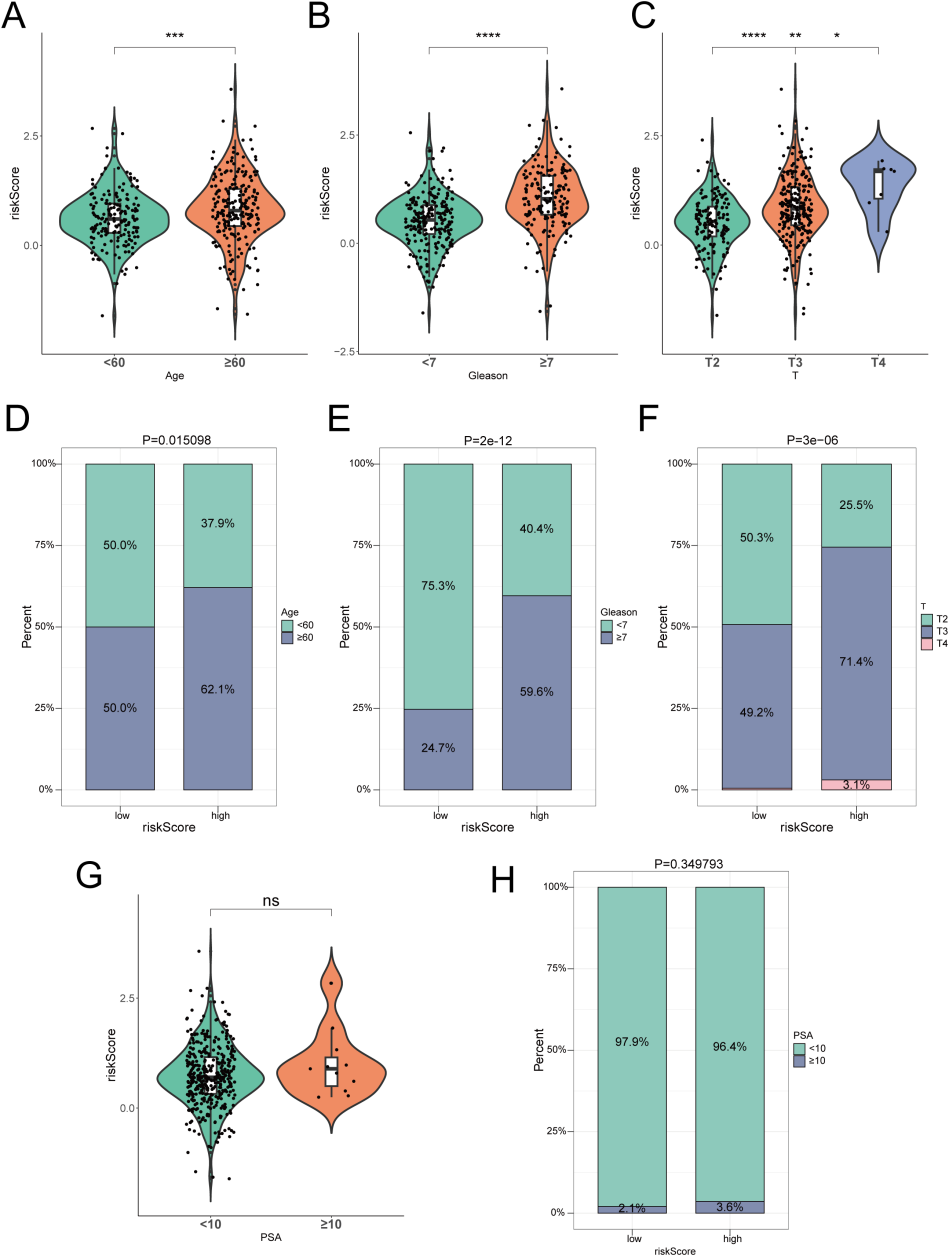
The clinical characteristics MPT-DN-RGs-related prognostic risk scores. **(A-C, G)** Violin Plot of Differences in the risk scores between **(A)** age groups (<60 vs. ≥60 years), **(B)** Gleason scores (<7 vs. ≥7), **(C)** T stages (T2, T3, T4), and **(G)** PSA value (<10 vs. ≥10). **(D-F, H)** Bar plots show the percentage distribution of **(D)** age (<60 vs. ≥60 years), **(E)** Gleason scores (<7 vs. ≥7), **(F)** T stages (T2, T3, T4), and **(H)** PSA value (<10 vs. ≥10) within low- and high-risk groups. Ns, not significant. * indicate p<0.05, ** indicate p<0.01, *** indicate p<0.001, **** indicate p<0.0001.

Analysis of risk scores across various clinical subtypes indicated that patients aged 60 years and older had significantly higher risk scores compared to those younger than 60 (p< 0.05) (**Figure 3A**). Furthermore, individuals with Gleason scores of 7 or higher exhibited substantially increased risk scores relative to those with scores below 7, while patients in stages T3 and T4 displayed notably higher risk scores than those in stage T2 (p< 0.05) (**Figures 3B, C**). Simultaneously, there were significant differences in the distribution proportions of HRG and LRG among clinical subtypes (p< 0.05), such as age, Gleason, and T stage (**Figures 3D–F**). These findings indicated that individuals aged 60 years and older, with Gleason scores of 7 or higher, and those at T3 and T4 stages might exhibit greater disease severity and potentially poorer prognosis. In contrast, no significant difference in risk scores was found between patients with PSA levels of 10 or higher and those with levels below 10, nor were there significant differences in the distribution proportions of HRG and LRG (p > 0.05) (**Figures 3G, H**).

### 3.6 Constructing a robust nomogram

**Figure 4:**
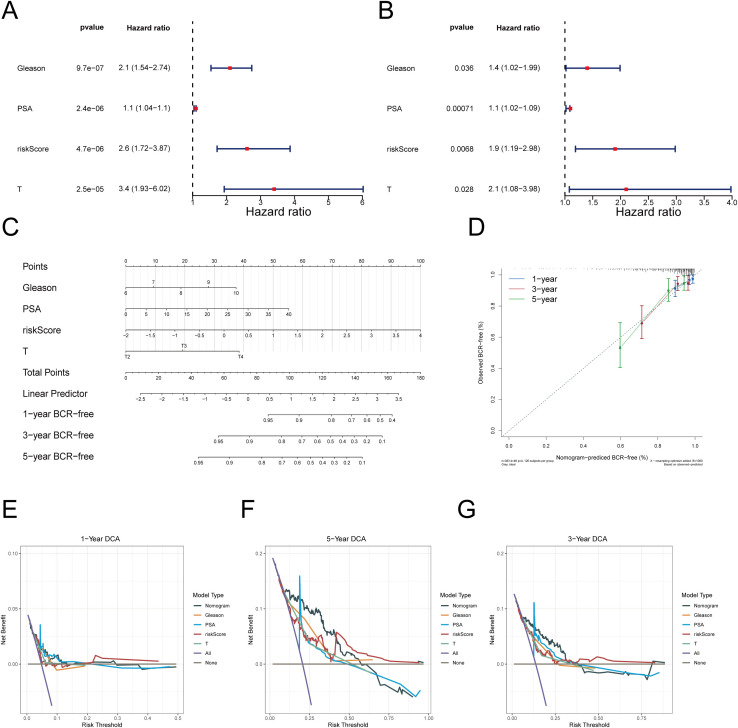
Construction and assessment of the nomogram survival model. **(A, B)** Univariate analysis and multivariate analysis for the clinical characteristics and risk scores. **(C)** Establishment of nomogram to predict the prognostic of PRAD patients. **(D)** Calibration plots showing the probability of 1-, 3-, and 5-year BCR-FS in TCGA-PRAD cohort. **(E-G)** Decision curve analysis (DCA) of nomogram predicting 1-**(E)**, 3-**(F)**, and 5-year **(G)** BCR-FS.

Subsequently, in the 396 PRAD samples of the training cohort, risk score and clinical characteristics of PRAD patients were sequentially entered into univariate Cox regression (HR ≠ 1, p< 0.05), PH assumption test (p > 0.05) and multivariate Cox regression (HR ≠ 1, p< 0.05) (**Figures 4A, B**; **Supplementary Table 4**). Eventually, the risk score, Gleason, T stage, and PSA were identified as independent prognostic factors. These factors were incorporated into a nomogram to predict the 1-, 3-, and 5-year BCR-FS probabilities of PRAD patients (**Figure 4C**). In this nomogram, each factor corresponded to an individual point, and the sum of these points yielded total points, which was used to predict the 1-, 3-, and 5-year BCR-FS probabilities for PRAD patients; the higher the total points, the lower the BCR-FS probability. Calibration curve indicated that the nomogram’s predicted probabilities for 1-, 3-, and 5-year BCR-FS closely align with the reference line, demonstrating high predictive accuracy (**Figure 4D**). DCA further demonstrated that the nomogram offered greater clinical utility than the use of any single independent prognostic factor alone (**Figures 4E–G**). These results signified the nomogram’s robust ability to assess prognosis in PRAD patients, although further validation in larger clinical samples was needed.

### 3.7 Exploring potential mechanisms of PRAD based on enrichment analysis

**Figure 5:**
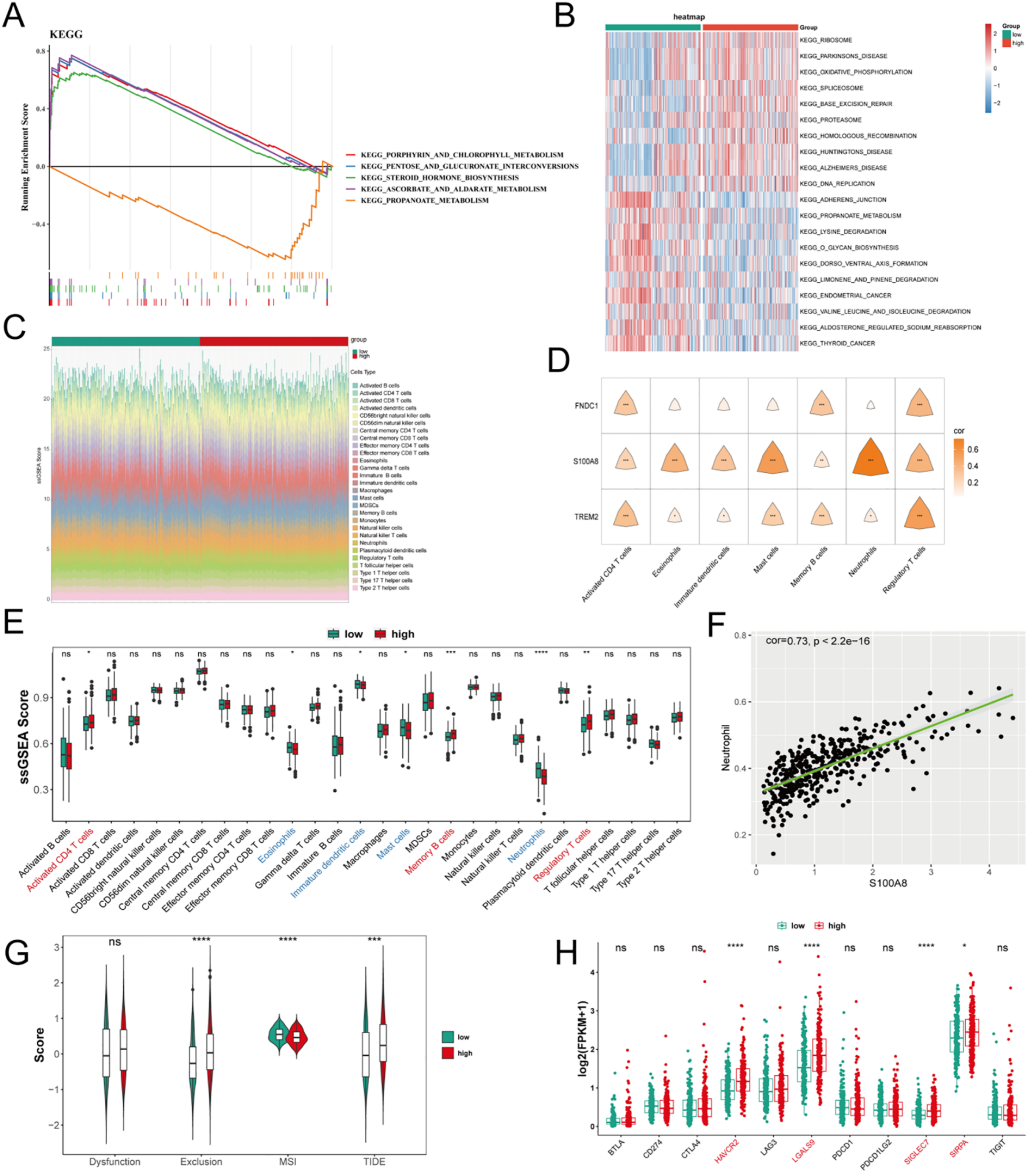
Enrichment analysis and immune-cell infiltration based on risk groups. **(A)** GSEA analysis showing top 5 pathways between low- and high-risk groups. **(B)** Heatmap of differences in pathways between low- and high-risk groups. **(C)** Stacked bar chart showing infiltration proportions of immune cells using ssGSEA. **(D)** Correlation analysis of FNDC1, S100A8, and TREM2 with differential immune infiltrating cells. **(E)** Box plot of differences in 7 types of immune cells within the low- and high-risk groups. **(F)** Correlation analysis between S100A8 and neutrophils. **(G)** The differences in the scores of dysfunction, exclusion, MSI, and TIDE between low- and high-risk groups. **(H)** The differences in the expression of immune checkpoint between low- and high-risk groups. Ns, not significant. * indicate p<0.05, ** indicate p<0.01, *** indicate p<0.001, **** indicate p<0.0001.

GSEA was conducted for the 2 identified risk groups, the analysis showcased that 15 KEGG pathways were significantly enriched (**Supplementary Table 5**). Notably, among the top 5 pathways, the samples in LRG were related to “propanoate metabolism”, as well as the pathways “pentose and glucuronate interconversions”, “porpyhrin and chlorophyll metabolism”, “ascorbate and aldarate metabolism”, and “steroid hormone biosynthesis” were enriched in HRG samples (**Figure 5A**). Moreover, this study revealed significant differences in pathways between HRG and LRG. For instance, pathways such as “adherens junction”, “lysine degradation”, and “limonene and pinene degradation” were suppressed in HRG (adj.p< 0.05), while pathways including “ribosome”, “oxidative phosphorylation”, “oxidative phosphorylation”, and “spliceosome” were activated in HRG (adj.p< 0.05) (**Figures 5B**; **Supplementary Table 5**). These findings might provide crucial insights into the underlying biological mechanisms that contribute to reduced risk in PRAD.

### 3.8 Dissecting the immunological landscapes across risk groups

Infiltration proportions of immune cells between HRG and LRG were further analyzed. Initially, the ssGSEA scores of 28 immune infiltrating cells in both types of samples were visualized within the HRG and LRG (**Figure 5C**), and box plot further visualized these differences, identifying significant disparities in 7 types of immune infiltrating cells (p< 0.05) (**Figure 5E**). Among them, activated CD4 T cells, memory B cells, and regulatory T cells showed significantly higher infiltration levels in HRG (p< 0.05), suggesting a more active cell-mediated immune response in HRG that might be linked to the tumor’s immune evasion mechanisms and the complex interactions within the tumor microenvironment. Conversely, eosinophils, mast cells, immature dendritic cells, and neutrophils exhibited significantly lower infiltration in HRG (p< 0.05), indicating a potential restriction in their regulatory or immunosuppressive functions within HRG. Furthermore, correlation analysis demonstrated that 3 prognostic genes were significantly positively correlated with the most of differential immune infiltrating cells (**Figures 5D**; **Supplementary Table 6**), with S100A8 exhibiting the strongest correlation with neutrophils (cor = 0.73, p*<* 0.001) (**Figure 5F**). Furthermore, TIDE and exclusion scores were significantly higher in the HRG group, whereas the MSI score exhibited a decrease (**Figure 5G**). There were no significant difference in dysfunction score between the 2 groups. Lastly, the analysis of differential immune checkpoint expression between HRG and LRG in PRAD revealed that HAVCR2, LGALS9, SIGLEC7, and SIRPA were significantly up-regulated in the HRG (p< 0.05) (**Figure 5H**). To characterize the immune landscape across risk strata, TIDE analysis was also performed on patients categorized into high-risk (HRG, n=198) and low-risk groups (LRG, n=198). The HRG exhibited significantly higher overall TIDE scores (Wilcoxon rank-sum test, p< 0.05), indicating a more pronounced immune-evasive phenotype. Interestingly, this elevation was primarily driven by increased immune exclusion scores rather than T-cell dysfunction (**Supplementary Figure 6**). These results suggest that immunosuppression in high-risk tumors is predominantly mediated by impaired immune cell infiltration into the tumor core (exclusion) rather than intrinsic T-cell exhaustion. This observation could impact the efficacy of some immune checkpoint inhibitors (ICIs), suggesting that high expression might serve as an indicator of poor prognosis for HRG.

### 3.9 Unraveling the mutational patterns and drug sensitivity

**Figure 6:**
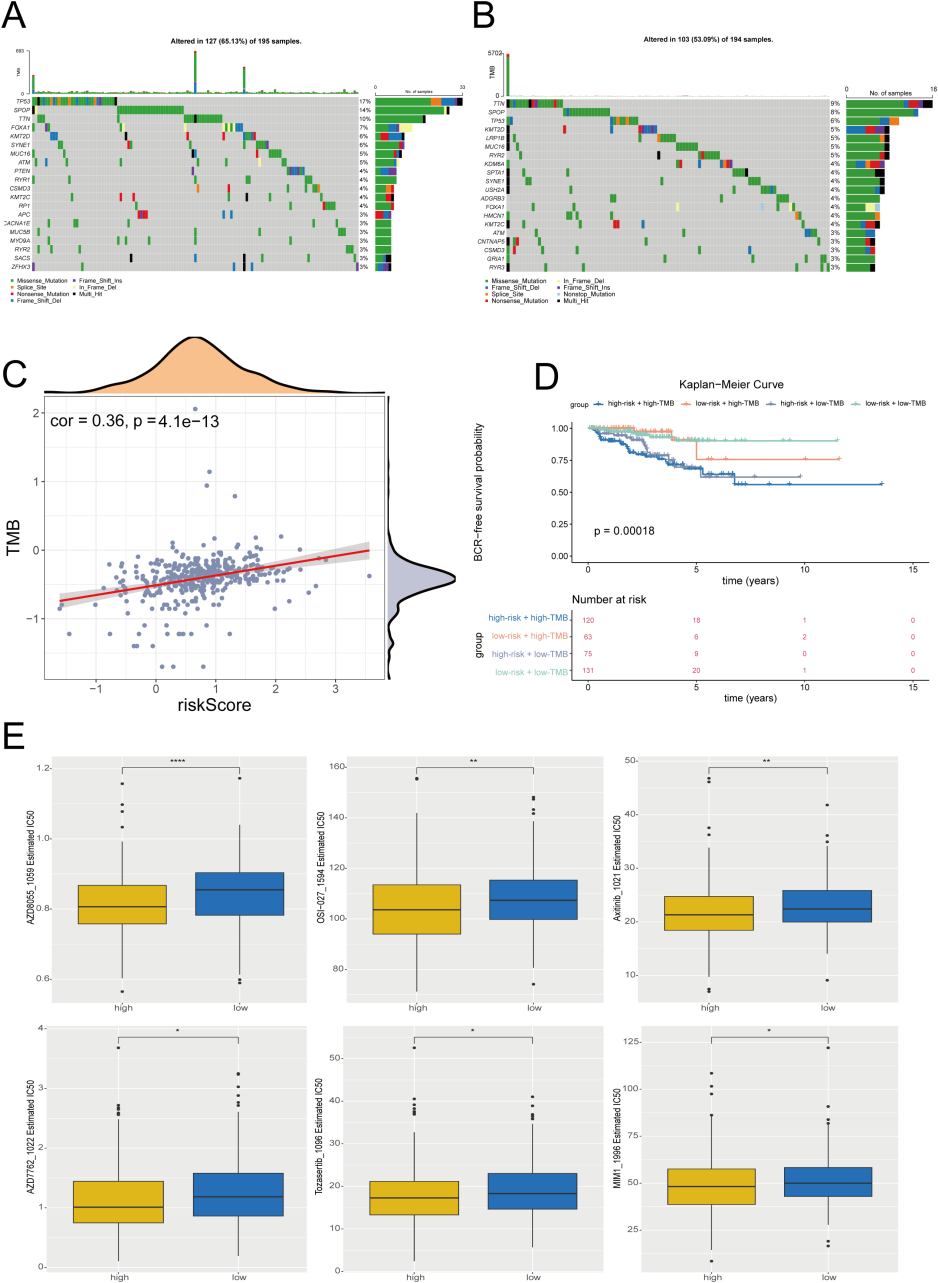
Mutational patterns and drug sensitivity. **(A, B)** Somatic mutation in high-risk group **(A)** and low-risk group **(B)**. **(C)** Correlation analysis between risk scores and TMB scores in PRAD patients. **(D)** Kaplan-Meier survival curves of high-risk + high-TMB, low-risk + high-TMB, high-risk + low-TMB, and low-risk + low-TMB. **(E)** Boxplots of the comparison of IC50 of drugs between high- and low-risk groups. * indicate p<0.05, ** indicate p<0.01, **** indicate p<0.0001.

The tumor mutational burden (TMB) was a quantifier of the total amount of genetic mutations present within tumor cells. To deepen the understanding, the somatic mutation profiles were explored across the 2 identified risk groups. It was noted that both groups predominantly exhibited missense mutations, with the TP53 showing the highest frequency of mutation in HRG and TTN in LRG (**Figures 6A, B**). Correlation analysis revealed a significant positive correlation between TMB scores and risk scores in 389 PRAD patients (cor = 0.36, p< 0.0001) (**Figure 6C**). Furthermore, an intriguing disparity was uncovered when comparing the 4 groups: HRG + high-TMB, HRG + low-TMB, LRG + high-TMB, and LRG + low-TMB, indicating a significant difference in BCR-FS (p = 0.00018) (**Figure 6D**). Additionally, analysis of drug sensitivity revealed significant differences in IC_50_ values across 2 risk groups for a total of 6 drugs, including AZD8055 1059, OSI-027 1594, Axitinib 1021, AZD7762 1022, Tozasertib 1096, and MIM1 1996, with all IC_50_ values being lower in HRG (p< 0.05) (**Figure 6E**). This might indicate that a lower concentration of the drug was required to inhibit 50% of the target, which implied higher potency.

### 3.10 Observation of prognostic genes expression

**Figure 7:**
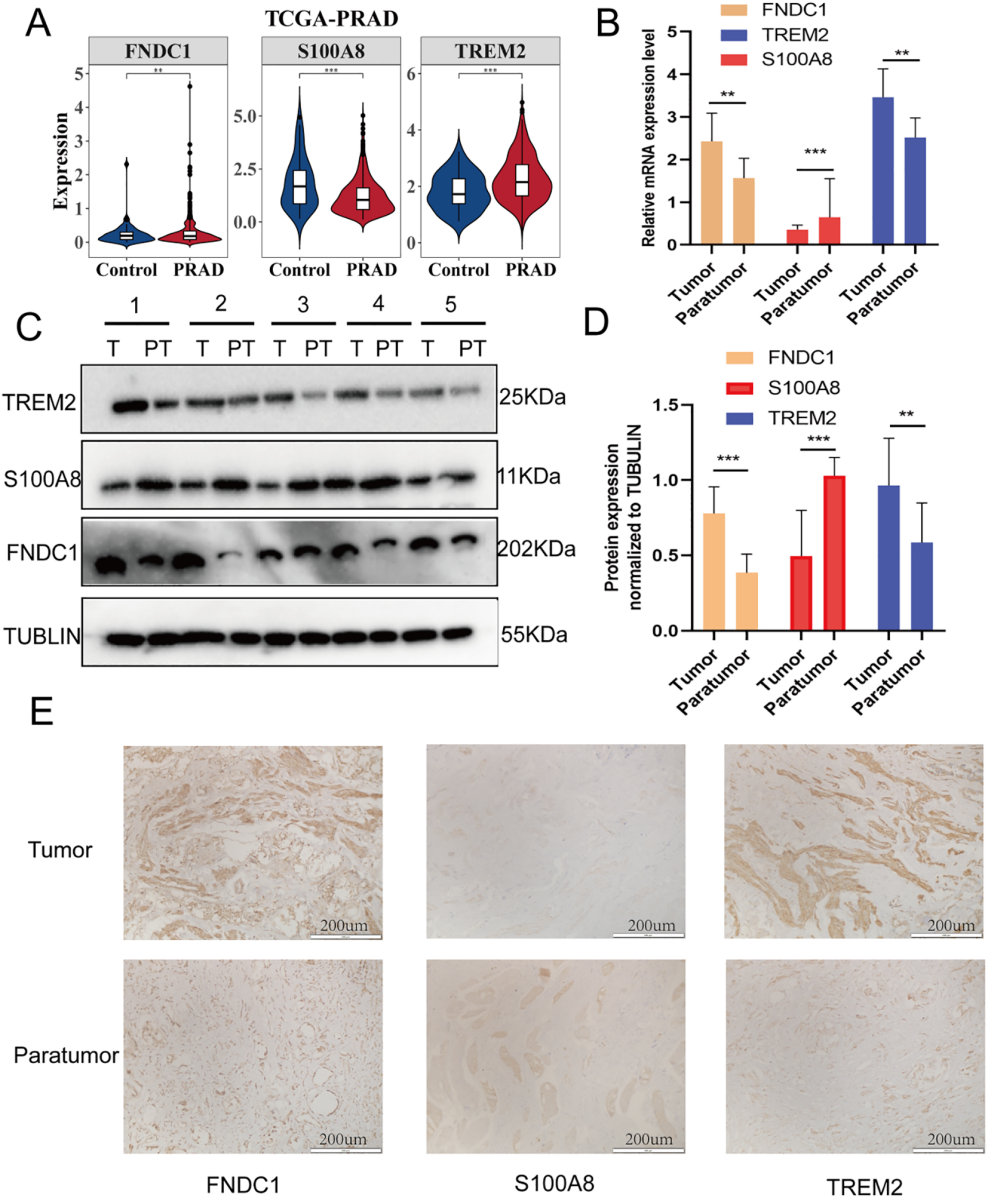
Validation of FNDC1, S100A8, and TREM2 expression. **(A)** The mRNA expression levels of FNDC1, S100A8, and TREM2 in TCGA-PRAD cohort. **(B)** The mRNA expression levels of FNDC1, S100A8 and TREM2 were confirmed by q-PCR between paratumor(PT) and prostate cancer tissue. **(C, D)**The protein expression levels of FNDC1, S100A8 and TREM2 were confirmed by western blot between paratumor(PT) and prostate cancer tissue(T). **(E)** Immunohistochemistry micrographs revealing FNDC1, S100A8 and TREM2 expression between paratumor(PT) and prostate cancer tissue(T). **P< 0.01; ***P< 0.001.

Analysis of TCGA database revealed that among PRAD samples, the prognostic genes TREM2 and FNDC1 demonstrated significant upregulation, while S100A8 showed marked downregulation in tumor tissues (**Figure 7A**). Moreover, data from the HPA database indicated that TREM2 and FNDC1 protein levels were upregulated in PRAD tumor tissues, whereas the expression of S100A8 protein was diminished (**Supplementary Figures 4A–C**). Furthermore, our validation also revealed that both TREM2 and FNDC1 exhibited high mRNA and protein expression in prostate cancer tissues, whereas S100A8 showed low expression in the same context (**Figures 7B–E**). These findings were consistent with results from TCGA and HPA databases.

## 4 Discussion

Biochemical recurrence (BCR) of prostate cancer suggests the potential existence of minimal residual disease or early recurrence, serving as a critical warning signal for disease progression. BCR typically indicates that the disease is in a progressive state, with a poorer prognosis (39). If timely treatment measures are not taken, it may lead to more severe symptoms and complications, such as local recurrence and distant metastasis. Therefore, regular follow-up and examinations are essential for the early detection of BCR and timely adjustment of treatment plans. MPT-DN is a non-apoptotic form of cell death triggered by oxidative stress and cytosolic Ca^2+^ overload, which relies on cyclophilin D (CypD) (40). In oncology, MPT-DN demonstrates significant prognostic value and predictive potential for therapeutic responses, with established associations with clinical outcomes in multiple malignancies, including lung cancer, breast cancer, endometrial cancer, cervical cancer, and hepatocellular carcinoma (6, 8, 40–42). However, while research on MPT-DN has advanced in solid tumors, its mechanistic role and clinical implications in prostate adenocarcinoma progression remain largely unexplored.

This study explores the relationship between MPT-DN and prostate adenocarcinoma. Through systematic analysis, we successfully identified three key genes closely associated with PRAD prognosis and constructed a risk assessment model and a nomogram based on these genes, providing a reliable tool for predicting the risk of BCR in PRAD patients. Furthermore, this study elucidated significant biological differences between high-risk and low-risk groups through GSEA, immune microenvironment evaluation, genetic mutation profiling, and drug sensitivity testing. Additionally, we validated the protein expression levels of the prognosis-related genes, offering new theoretical and practical insights for PRAD prognosis assessment, clinical research, and therapeutic strategy development. We initially expanded the 39 MPT-DN-related genes to identify core module genes closely associated with the MPT-DN pathway in PRAD. Subsequently, we performed differential expression analysis comparing PRAD tissues with normal prostate tissues, as well as between patients with BCR and those without recurrence (no-BCR), resulting in two distinct sets of differentially expressed genes (DEGs1 and DEGs2). By intersecting these three gene sets, we ultimately narrowed down to 23 candidate genes. Through a combination of univariate Cox regression analysis, Lasso regression modeling, and multivariate Cox regression analysis, we identified three key genes significantly associated with PRAD prognosis: TREM2, FNDC1, and S100A8.

TREM2 is involved in the regulation of cellular energy metabolism and mitochondrial autophagy (43, 44). TREM2 can reduce the intracellular reactive oxygen species (ROS) levels, thereby alleviating the damage to mitochondrial membranes caused by oxidative stress (43, 45). Notably, ROS is considered one of the crucial triggers for the opening of the mitochondrial permeability transition pore (mPTP) (46). Based on this, TREM2 can mitigate mitochondrial dysfunction (MPT-DN) resulting from the opening of the mitochondrial permeability transition pore by regulating ROS levels. Mounting clinical evidence links TREM2 overexpression with accelerated neoplastic proliferation, metastatic dissemination, and aggressive clinical manifestations across multiple cancers—particularly hepatocellular carcinoma, glioma, and prostate malignancies (47–49). Mechanistic studies indicate TREM2 potentiates tumorigenesis through: Enhancing cancer cell survival pathways, activating proliferative signaling cascades and establishing permissive niches for metastasis (50). However, these tumor studies did not mention that TREM2 promotes tumor progression by alleviating MPT-DN. This also makes TREM2 a promising research target in tumors.

FNDC1 is a protein located on the outer mitochondrial membrane that is involved in mitochondrial quality control and cellular homeostasis regulation. It primarily affects the process of mitophagy, leading to the obstruction of the clearance of mitochondria that generate reactive oxygen species (ROS).This, in turn, exacerbates oxidative stress, which may subsequently impact the stability of the mitochondrial membrane and participate in the process of MPT-DN (51). Clinically, elevated FNDC1 expression shows a strong association with poorer prognosis in various cancers, such as lung adenocarcinoma, colorectal cancer, gastric cancer, pancreatic cancer, and prostate cancer (51–56). These findings highlight FNDC1’s potential as both a therapeutic target and a prognostic biomarker in oncology.

S100A8 is a crucial multifunctional protein belonging to the S100 protein family, playing a pivotal role in inflammation, immune responses, cytoskeletal regulation, and cancer progression (57, 58). S100A8 can induce mitochondrial respiratory dysfunction by increasing the production of reactive oxygen species (ROS), leading to mitochondrial membrane depolarization and the release of cytochrome from mitochondria into the cytoplasm, thereby triggering apoptosis and necrosis (59–62). Studies have revealed that the S100A8/A9 complex can induce excessive mitochondrial fission and impaired mitophagy in a Sirt1-dependent manner, resulting in the accumulation of damaged mitochondria and increased release of mitochondrial DNA (mtDNA). This ultimately activates ZBP1-mediated PANoptosis, a novel form of programmed cell death encompassing apoptosis, pyroptosis, and necrosis, suggesting a significant association between S100A8 and mitochondrial permeability-associated necrosis (61). Clinically, emerging evidence further implicates S100A8 in direct oncogenic mechanisms, with observed associations between its expression levels and tumor cell proliferation dynamics, survival signaling, and metastatic competence (63, 64). Notably, comparative analysis of prostate tissues reveals downregulated S100A8 expression in localized prostate adenocarcinoma (PRAD) compared to benign prostatic hyperplasia (BPH), while paradoxically observing upregulation in aggressive PRAD variants. This expression pattern correlates inversely with biochemical recurrence-free survival outcomes, suggesting dual utility: firstly as a discriminatory biomarker for malignant versus benign prostatic pathology, and secondly as a prognostic indicator of clinical aggressiveness and recurrence risk in established malignancies (65).

Although single-gene biomarkers have been utilized for prognostic evaluation and therapeutic response prediction in prostate cancer, their predictive accuracy remains suboptimal. In this study, we found that the risk scoring model derived from MPT-DN-related genes exhibited robust prognostic performance across both the training cohort (TCGA-PRAD) and an independently validated cohort (GSE70768). Notably, our MPT-DN signature comprises only three core genes (TREM2, FNDC1, and S100A8), whereas many previously reported models rely on more extensive gene sets. The limited number of genes may facilitate practical implementation and reduce assay complexity compared with larger panels. While achieving predictive performance comparable to these established models, the MPT-DN signature provides a more robust biological rationale, potentially facilitating its clinical translation and functional validation.

Further analysis revealed a significant synergistic effect between the risk score and conventional clinicopathological parameters, including Gleason score, T stage, and serum PSA levels. Notably, the proportion of patients with Gleason scores ≥8 (ISUP grade 4-5) was significantly higher in the high-risk group compared to the low-risk group, indicating the model’s efficacy in identifying individuals with aggressive clinicopathological characteristics. In addition to prognostic stratification, we explored potential therapeutic implications of the MPT-DN–based risk model by estimating drug sensitivity using pharmacogenomic data from cancer cell lines. Notably, patients in the high-risk group exhibited significantly lower predicted IC50 values for six agents, including AZD8055, OSI-027, Axitinib, AZD7762, Tozasertib, and MIM1. This phenomenon may be attributed to metabolic reprogramming-induced alterations in drug sensitivity and differential expression profiles of drug resistance-associated genes in tumor cells.

Previous studies have predominantly focused on the association between mitochondrial dysfunction and tumorigenesis and progression. For instance, OPA1 modulates tumor angiogenesis in tumor by regulating mitochondrial fusion dynamics (66), whereas DRP1-mediated mitochondrial fission is closely linked to enhanced tumor cell migratory capacity (67). However, the current study is the first to incorporate the MPT-DN-related genes as an integrated module into prognostic model development, while systematically validating its biological functions in regulating the tumor immune microenvironment. Notably, our findings align closely with single-cell sequencing analyses, revealing a prominent immunosuppressive phenotype in the high-risk group tumor microenvironment characterized by significantly increased infiltration of M2-type tumor-associated macrophages and suppressed functional activity of CD8+ cytotoxic T lymphocytes. While high-risk tumors exhibited elevated regulatory T-cell levels, TIDE analysis revealed that their immunosuppressive phenotype was primarily driven by immune exclusion rather than T-cell dysfunction. This suggests that immune evasion in high-risk patients is predominantly mediated by impaired immune cell infiltration into the tumor core—restricting access to effector cells—rather than intrinsic functional exhaustion of T cells. Consequently, “immunosuppression” in this context reflects a microenvironment characterized by exclusion-based evasion, providing a more precise mechanistic alignment between the prognostic risk model and the observed immunological landscape. This observation provides a novel mechanistic explanation for clinical immunotherapy resistance in prostate cancer, suggesting that MPT-DN may modulate immunotherapy efficacy through remodeling the composition of the tumor immune microenvironment.

This study employed a multi-omics integration strategy, complemented by machine learning algorithms and bioinformatics analyses, to identify three prognosis-related genes (TREM2, FNDC1, and S100A8) in prostate cancer. We elucidated the associations and correlations between these genes and PRAD prognosis, as well as their biological functions in the progression of PRAD. We innovatively developed a prognostic assessment system based on the MPT-DN, overcoming the limitations of traditional single-gene analyses by integrating gene expression profiles with clinicopathological parameters, thereby significantly enhancing predictive accuracy. Further analyses demonstrated that this risk model effectively reflects tumor immune microenvironment characteristics and chemosensitivity profiles, providing critical theoretical support for personalized therapeutic strategies in PRAD.

Regarding the mechanistic connection between the identified genes (TREM2, FNDC1, and S100A8) and MPT-DN pathways, several hypotheses can be proposed. While our study primarily identified associations and correlations, further research is needed to determine whether these genes directly regulate MPT-DN opening or act as downstream effectors in MPT-DN pathways. It is also possible that the association is indirect, occurring through immune or metabolic reprogramming within the tumor microenvironment. For instance, TREM2, known for its role in immune regulation, may influence MPT-DN indirectly by modulating the tumor immune landscape. Similarly, FNDC1 and S100A8 may participate in metabolic reprogramming, affecting mitochondrial function and, consequently, MPT-DN. Future functional studies are warranted to elucidate these mechanistic connections. Besides, several limitations warrant attention. First, as this study relied primarily on publicly available datasets, prospective validation in large-scale multicenter cohorts is required to further confirm the clinical applicability of the proposed model. Second, the number of BCR events in the training cohort was relatively limited compared with the multi-step feature selection procedure, which may increase the risk of model overfitting. Although LASSO regression and external validation were applied to mitigate this risk, prospective validation in larger independent cohorts is still warranted. Third, the regulatory networks and precise functional mechanisms of MPT-DN-related genes remain incompletely understood, and further experimental investigations, including gene editing and organoid-based models, are needed to clarify their biological roles. In addition, the drug sensitivity analysis was derived from cell line–based pharmacogenomic databases, which may not fully recapitulate the complexity of clinical treatment responses. Therefore, these findings should be interpreted as exploratory and hypothesis-generating rather than direct evidence of clinical efficacy. Moreover, potential confounding factors such as ethnicity, geographic variation, and environmental exposures were not considered, which may limit the generalizability of the model across diverse populations. Future studies should prioritize multinational prospective clinical validation and integrate emerging technologies such as single-cell sequencing to dynamically characterize the evolutionary expression patterns of MPT-DN-related genes during tumor progression.

## 5 Conclusions

This study identified three prognosis-related genes (TREM2, FNDC1, and S100A8) in prostate cancer and developed a prognostic assessment system based on MPT-DN-related genes. By integrating gene expression profiles with clinicopathological parameters, we significantly enhanced predictive accuracy compared to traditional single-gene analyses. Further analyses demonstrated that this risk model effectively reflects tumor immune microenvironment characteristics and chemosensitivity profiles, providing critical theoretical support for personalized therapeutic strategies in PRAD.

## Data Availability

The datasets presented in this study can be found in online repositories. The names of the repository/repositories and accession number(s) can be found in the article/**Supplementary Material**.

## Supplementary material

Supplementary Figure 1 Identification of MPT-DN-RGs-related candidate genes through integrated WGCNA and differential expression analysis. **(A)** Sample dendrogram and trait heatmap showing clustering patterns and MPT-DN-RGs score distribution. **(B)** Scale independence (left) and mean connectivity (right) as functions of soft-thresholding power. **(C)** Gene clustering dendrogram with dynamically assigned module colors. **(D, E)** Heatmaps of differentially expressed genes (DEG1 and DEG2) across groups (Control vs. PRAD, and no-BCR vs. BCR), with expression values scaled by row.

Supplementary Figure 2 Kaplan-Meier survival analysis of FNDC1, S100A8, and TREM2 expression groups. **(A-L)** Kaplan-Meier survival analysis showing difference in DFS, OS, PFS, and RFS between high- and low-expression groups of FNDC1 **(A, D, G, J)**, S100A8 **(B, E, H, K)**, and TREM2 **(C, F, I, L).**

Supplementary Figure 3 Training and validation of the gene signature prediction model. **(A, C)** Distribution of risk scores according to the BCR status and time in TCGA train set **(A)** and GSE70768 validation set **(C)**. **(B, D)** Heatmap of TREM2, FNDC1, and S100A8 genes in TCGA train set **(B)** and GSE70768 validation set **(D)**. **(E)** Time‐dependent ROC curve analysis of risk score at 1-, 3-, and 5-years BCR-FS time in GSE70768 validation set.

Supplementary Figure 4 The protein expression levels. FNDC1 **(A)**, S100A8 **(B)**, and TREM2 **(C)** were confirmed by the HPA database between normal tissue and prostate cancer tissue(PCa).

Supplementary Figure 5 Comparison of predictive performance between the MPT-DN signature and previously reported multigene models for PRAD BCR. **(A)** Decision curve analysis showing the clinical net benefit of different prognostic models across various threshold probabilities. **(B)** Comparison of C-index and time-dependent ROC curves evaluating the predictive accuracy of different models for BCR-FS in the validation cohort.

Supplementary Figure 6 Comparison of TIDE scores and immune evasion characteristics between high- and low-risk groups.
