# Supplementary figures and images for: A novel prognostic signature based on mitochondrial permeability transition-driven necrosis genes for biochemical recurrence prediction in prostate cancer
Source: Front Oncol. 2026 Apr 2;16:1775602. doi: 10.3389/fonc.2026.1775602 (PMC13082966; doi:10.3389/fonc.2026.1775602)

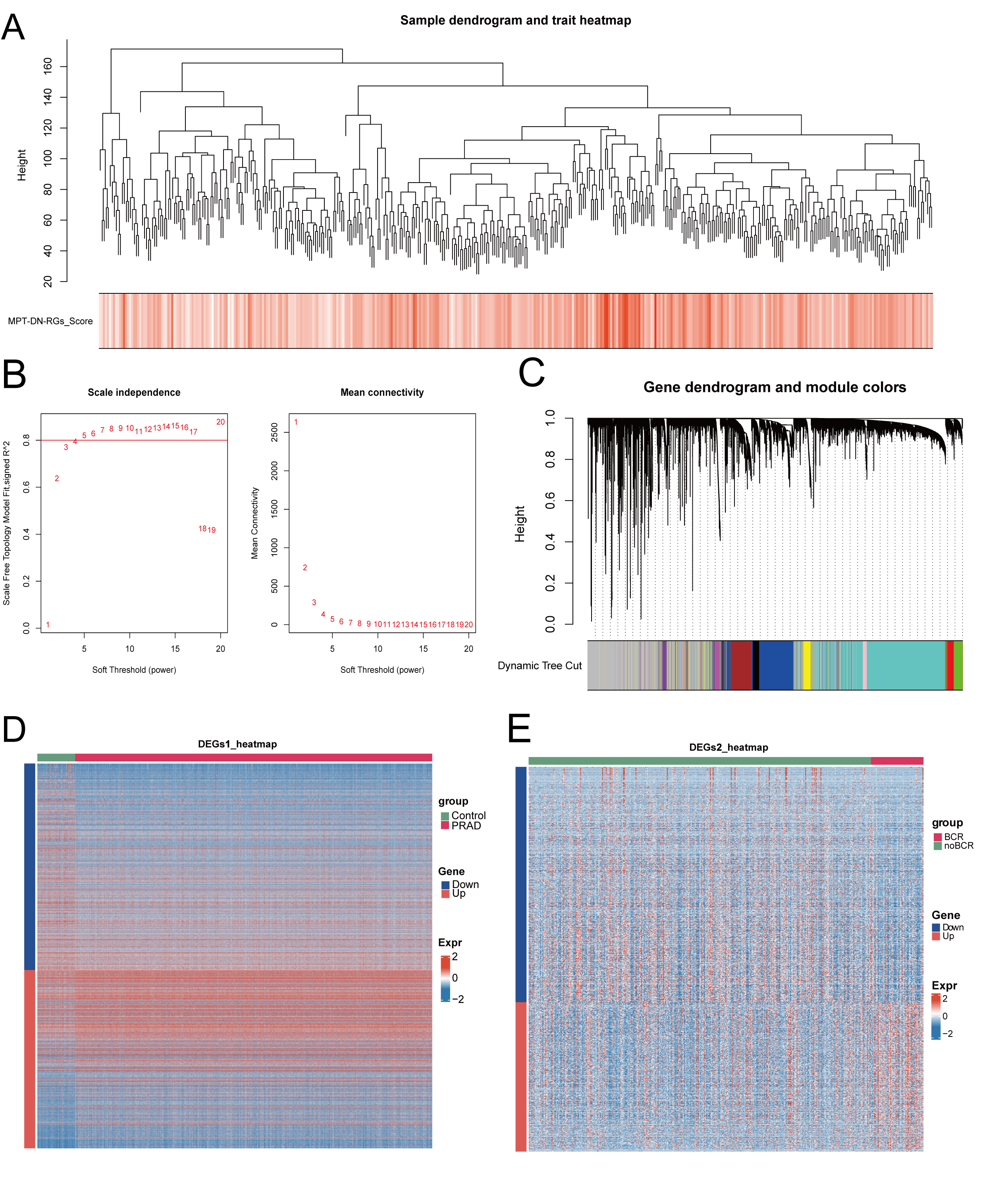

Supplement: Supplementary Figure 1 — Identification of MPT-DN-RGs-related candidate genes through integrated WGCNA and differential expression analysis. (A) Sample dendrogram and trait heatmap showing clustering patterns and MPT-DN-RGs score distribution. (B) Scale independence (left) and mean connectivity (right) as functions of soft-thresholding power. (C) Gene clustering dendrogram with dynamically assigned module colors. (D, E) Heatmaps of differentially expressed genes (DEG1 and DEG2) across groups (Control vs. PRAD, and no-BCR vs. BCR), with expression values scaled by row. [file Image1.tif]

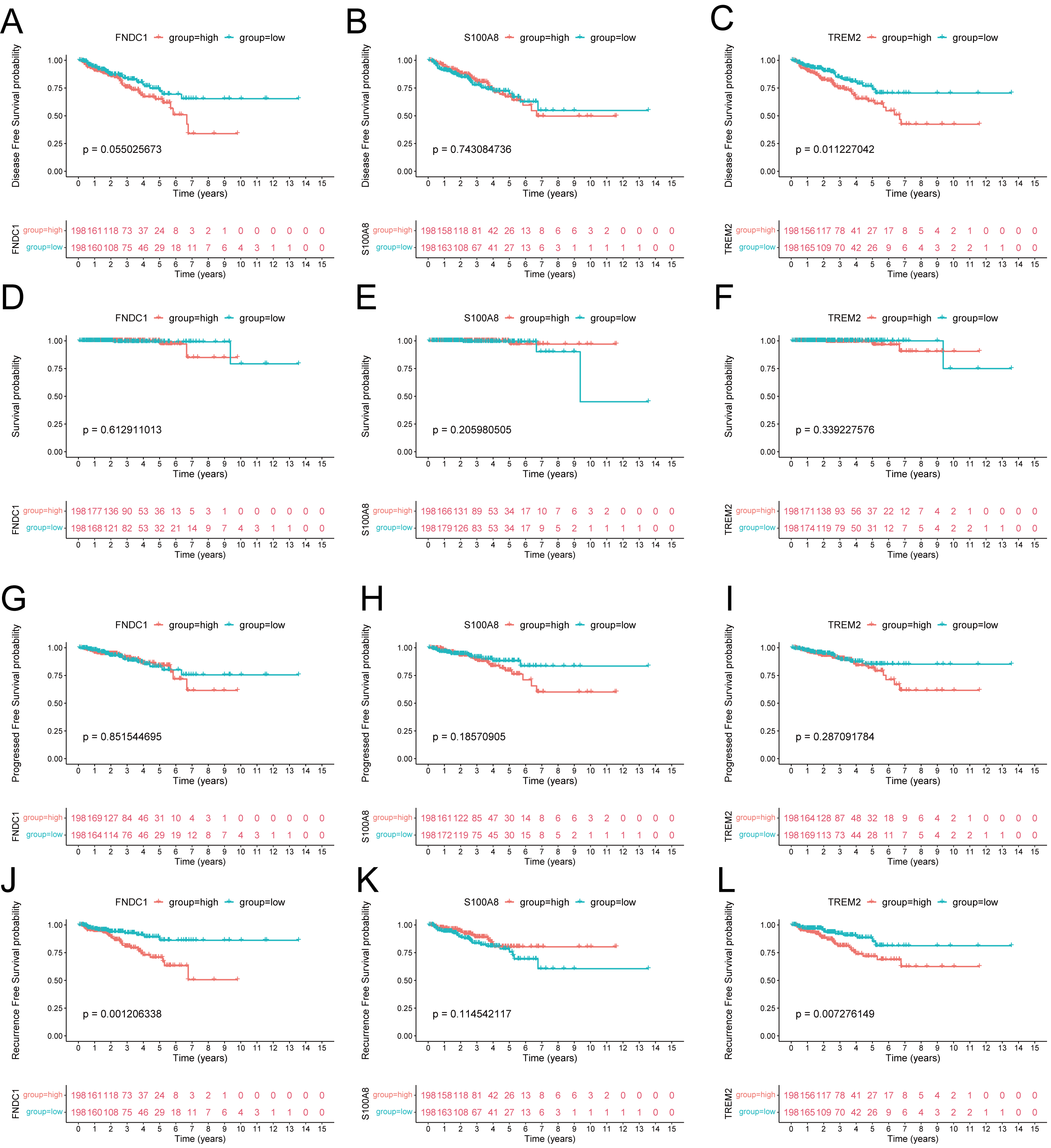

Supplement: Supplementary Figure 2 — Kaplan-Meier survival analysis of FNDC1, S100A8, and TREM2 expression groups. (A-L) Kaplan-Meier survival analysis showing difference in DFS, OS, PFS, and RFS between high- and low-expression groups of FNDC1 (A, D, G, J), S100A8 (B, E, H, K), and TREM2 (C, F, I, L). [file Image2.tif]

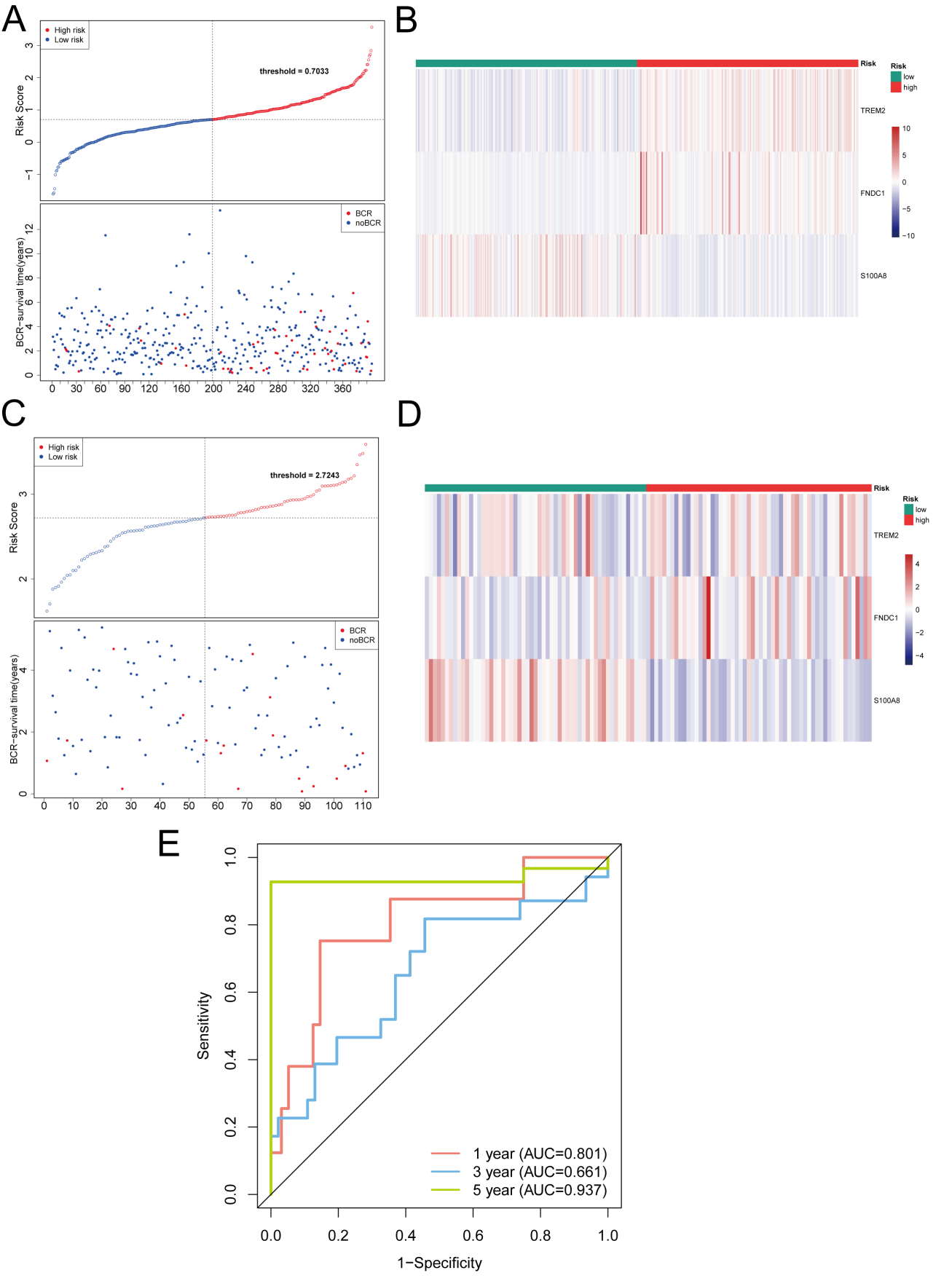

Supplement: Supplementary Figure 3 — Training and validation of the gene signature prediction model. (A, C) Distribution of risk scores according to the BCR status and time in TCGA train set (A) and GSE70768 validation set (C). (B, D) Heatmap of TREM2, FNDC1, and S100A8 genes in TCGA train set (B) and GSE70768 validation set (D). (E) Time‐dependent ROC curve analysis of risk score at 1-, 3-, and 5-years BCR-FS time in GSE70768 validation set. [file Image3.tiff]

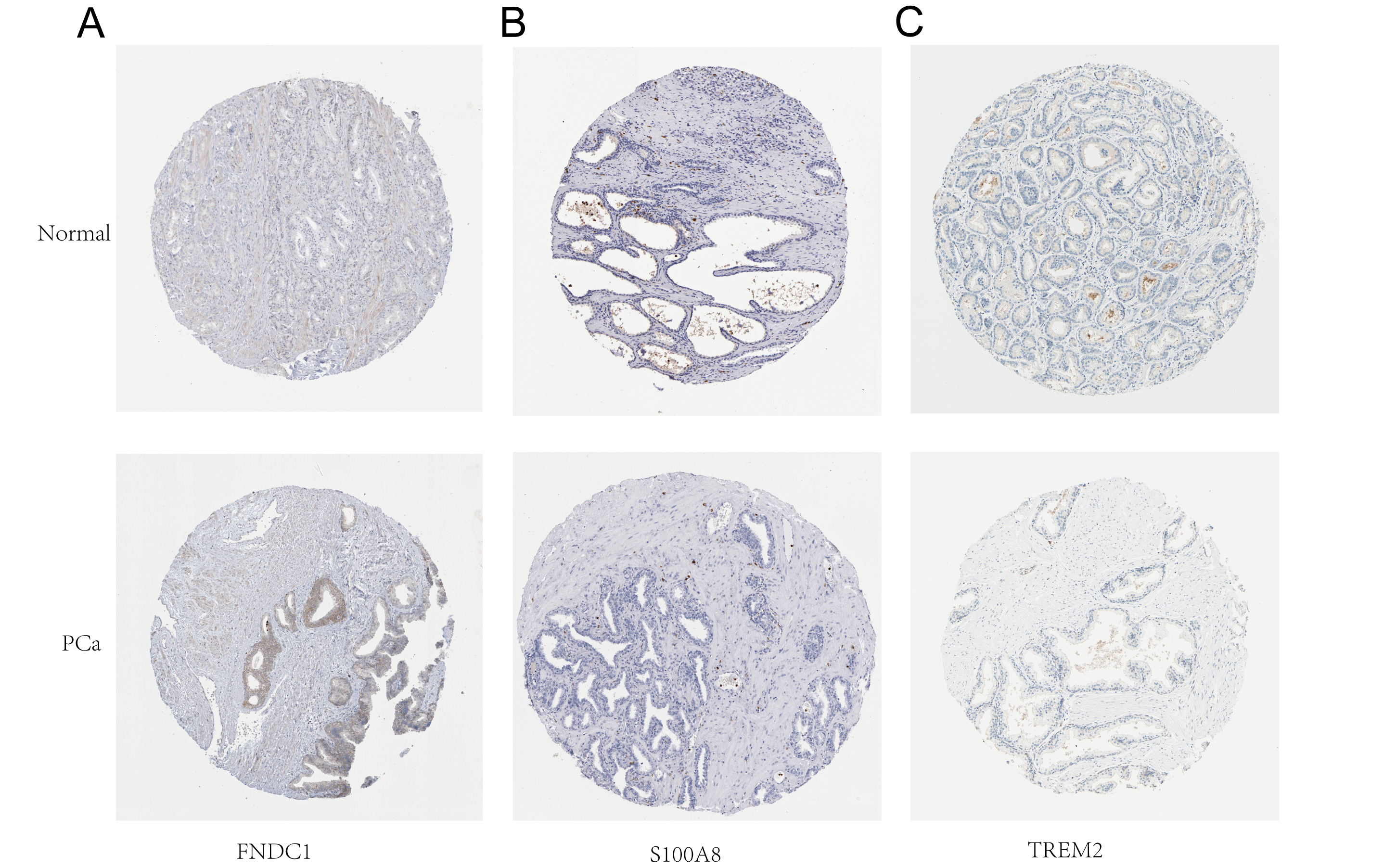

Supplement: Supplementary Figure 4 — The protein expression levels. FNDC1 (A), S100A8 (B), and TREM2 (C) were confirmed by the HPA database between normal tissue and prostate cancer tissue(PCa). [file Image4.tif]

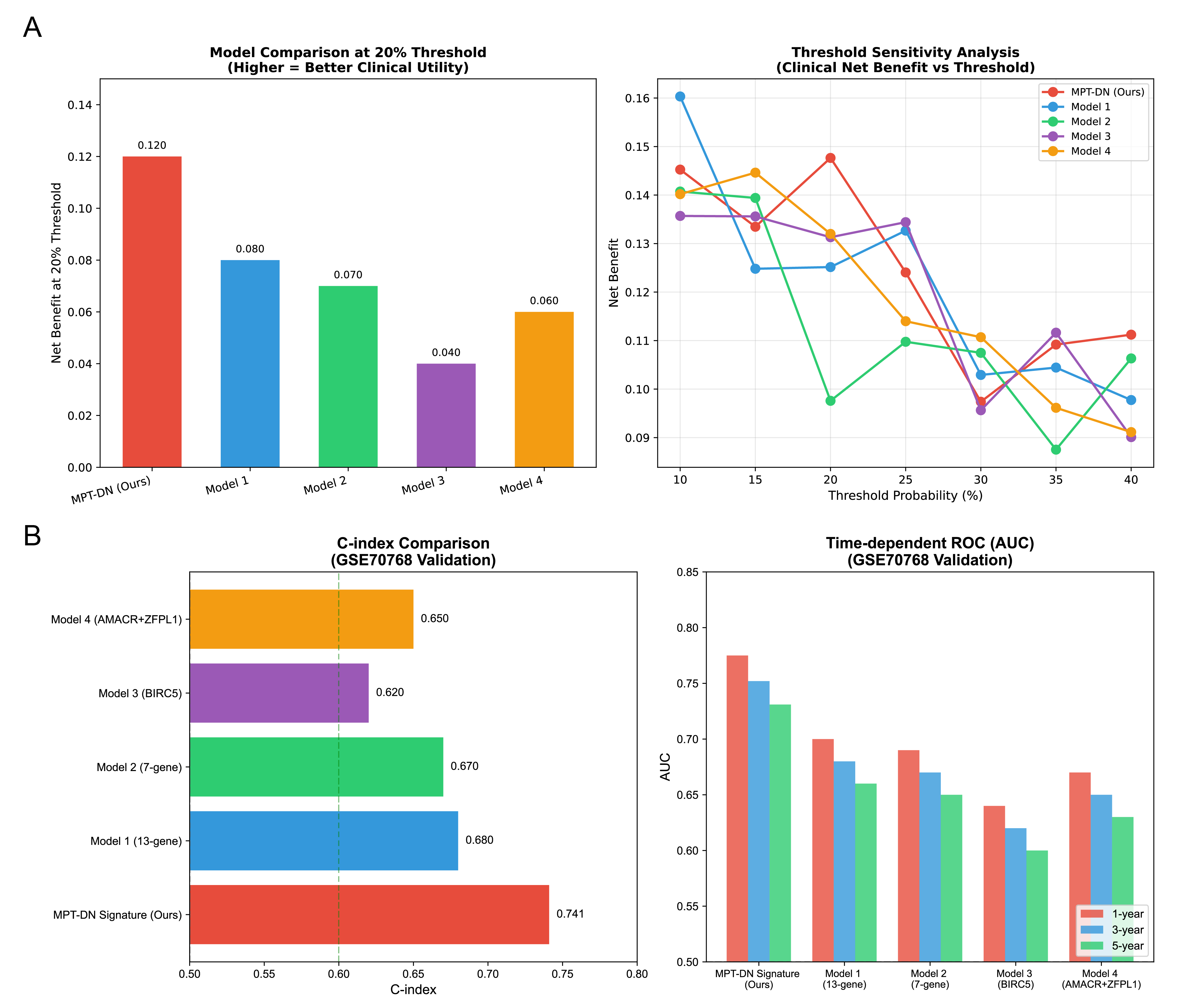

Supplement: Supplementary Figure 5 — Comparison of predictive performance between the MPT-DN signature and previously reported multigene models for PRAD BCR. (A) Decision curve analysis showing the clinical net benefit of different prognostic models across various threshold probabilities. (B) Comparison of C-index and time-dependent ROC curves evaluating the predictive accuracy of different models for BCR-FS in the validation cohort. [file Image5.tif]

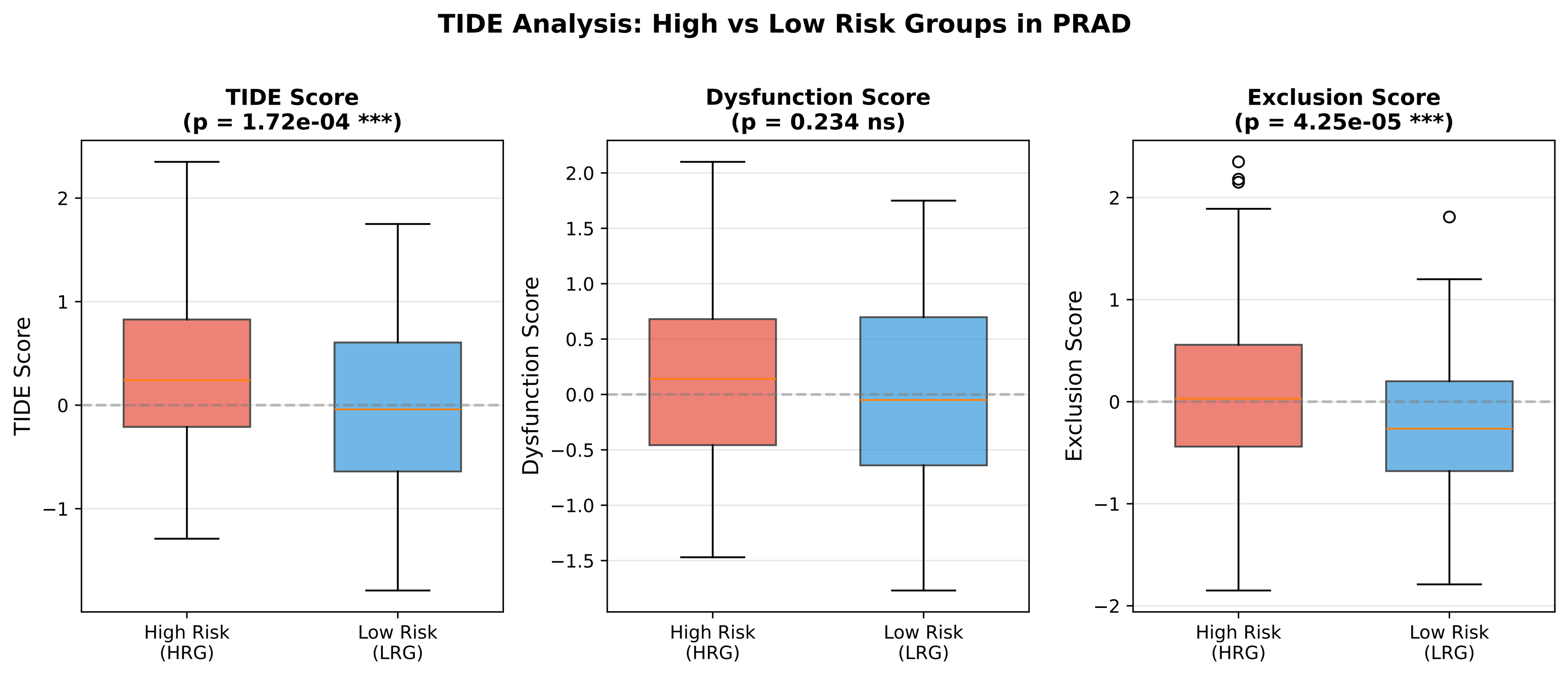

Supplement: Supplementary Figure 6 — Comparison of TIDE scores and immune evasion characteristics between high- and low-risk groups. [file Image6.tiff]
